# Collagen Type IV Variants and Kidney Cysts: Decoding the COL4A Puzzle

**DOI:** 10.3390/genes16060642

**Published:** 2025-05-27

**Authors:** Matteo Rigato, Carlotta Caprara, J. Said Cabrera-Aguilar, Nenzi Marzano, Anna Giuliani, Barbara Mancini, Fiorella Gastaldon, Claudio Ronco, Monica Zanella, Daniela Zuccarello, Valentina Corradi

**Affiliations:** 1 Department of Nephrology, Dialysis and Transplantation, AULSS8 BERICA, San Bortolo Hospital, 36100 Vicenza, Italy; matteo.rigato@aulss8.veneto.it (M.R.); carlotta.caprara@aulss8.veneto.it (C.C.); anna.giuliani@aulss8.veneto.it (A.G.); fiorella.gastaldon@aulss8.veneto.it (F.G.); monica.zanella@aulss8.veneto.it (M.Z.); valentina.corradi@aulss8.veneto.it (V.C.); 2 The International Renal Research Institute of Vicenza (IRRIV) Foundation, AULSS8 BERICA, San Bortolo Hospital, 36100 Vicenza, Italy; josesaidcabrera@gmail.com (J.S.C.-A.); nenzimarzano@yahoo.it (N.M.); cronco@goldnet.it (C.R.); 3 Department of Medical Genetics and Genomics, AULSS8 BERICA, San Bortolo Hospital, 36100 Vicenza, Italy; barbara.mancini@aulss8.veneto.it; 4 Nephrology Service, Hospital Civil de Guadalajara Fray Antonio Alcalde, Guadalajara 44200, Mexico

**Keywords:** cystic kidney disease, *COL4A*, collagen variants, kidney cysts, Alport syndrome, ADPKD

## Abstract

Pathogenic variants in type IV collagen genes (*COL4A3*, *COL4A4*, *COL4A5*) are classically associated with Alport syndrome (AS), a hereditary nephropathy primarily affecting the glomerular basement membrane (GBM). Recent findings, however, suggest a broader phenotypic spectrum that includes renal cyst formation, raising questions about overlapping mechanisms with other cystic kidney diseases. Clinically, renal cysts have been increasingly reported in patients with autosomal dominant and X-linked forms of Alport syndrome, particularly in association with glycine missense variants. The most recent studies focusing on the cystic phenotype in Alport syndrome provide growing support for the idea that variants in type IV collagen genes are associated with an increased likelihood of developing renal cysts, likely through mechanisms involving the structural integrity of renal basement membranes. In this review, we explore evidence from murine models and human studies indicating defects in collagen IV and discuss their contribution to cystogenesis. These observations underscore the need for broader genetic screening strategies and further investigation into the molecular mechanisms underlying this emerging phenotype.

## 1. Introduction

Renal cysts, although often benign and asymptomatic, can present in various forms with differing implications for renal function. The growth of small, simple cysts is typically about 1 mm per year [1]. However, it has been observed that larger cysts tend to grow at a faster rate [2]. These cysts may be found incidentally in imaging studies and commonly grow proportionally over time [3].

The presence of cysts in renal tubules is a common feature in various kidney diseases, both genetic and acquired. Although autosomal dominant polycystic kidney disease (ADPKD) is the most frequent cause of polycystic kidneys, other conditions may also present renal cysts as part of their phenotype, such as Alport syndrome (AS), as shown in Table 1.

ADPKD is characterized by the formation and expansion of cysts in the renal tubules, ultimately leading to end-stage chronic kidney disease. The new guidelines [4] classify the causative genes of ADPKD into different categories: *major genes* (*PKD1* and *PKD2*, the causative genes in >95% of typical ADPKD cases with a risk of kidney failure); *minor genes* (*ALG5*, *ALG9*, *DNAJB11*, *GANAB*, *IFT140*, *NEK8*, and *PKHD1*) with strong supporting data of pathogenicity; and *suggested genes*, for which supporting data are limited at this stage. Variations in *PKD1* are generally associated with a more severe disease course and an earlier onset of kidney failure compared to those in *PKD2*. According to the genetic threshold model, a critical reduction in the expression of these genes leads to cyst formation [5]. 

Alport syndrome is the second most common monogenic cause of chronic kidney disease (CKD). This syndrome is caused by pathogenic variants in the *COL4A5*, *COL4A4*, and *COL4A3* genes, which encode the α5(IV), α4(IV), and α3(IV) collagen type IV chains, respectively, in that order of frequency. In AS, although the primary manifestation involves the glomerulus with abnormalities of the glomerular basement membrane (GBM), cystic kidney disease can also be observed in affected individuals. Pathogenic variants in *COL4A3*, *COL4A4*, and *COL4A5* lead to structural defects in the GBM, often resulting in the absence or reduction of α3(IV), α4(IV), and α5(IV) chains and their replacement by α1(IV) and α2(IV) chains [6].

Type IV collagen is a major structural component of GBM. In vertebrates, it is encoded by six genes, designated *COL4A1-6*. These genes encode six distinct α(IV) collagen chains, which assemble into three distinct triple-helical trimers: α1α1α2 (IV), α3α4α5(IV), and α5α5α6(IV). Each α(IV) chain contains a non-collagenous domain at the N-terminus, a central collagenous domain rich in Gly-X-Y repeats, and a C-terminal non-collagenous (NC1) domain [7]. 

The distribution of these different α(IV) chains varies within the kidney. In the mature human and rodent kidney, α1(IV) and α2(IV) chains are expressed at low levels in the GBM but are highly expressed in tubular basement membranes (TBMs) and the Bowman’s capsule [8]. Conversely, the α3(IV), α4(IV), and α5(IV) chains are predominantly expressed in the mature GBM and TBMs. The GBM is formed by the fusion of separate podocyte and endothelial BMs during glomerulogenesis [9].

The abnormalities of the TBMs in these cases have been described as reminiscent of the “basket-weave” appearance seen in the GBM in AS. This highlights that defects in type IV collagen, depending on the affected chains and their tissue distribution, can cause diffuse pathology of the renal basement membrane.

Thin basement membrane disease (TBMD), characterized by microscopic hematuria, typically with normal kidney function and the absence of proteinuria, is a common hereditary renal disease characterized by a diffuse thinning of the GBM. It is caused by heterozygous pathogenic variants in *COL4A3* and *COL4A4*. However, this term has largely fallen out of use, as the histological finding of GBM thinning is now considered a manifestation of AS rather than a separate entity.

Historically, women with X-linked AS, due to the X chromosome inactivation phenomenon, were considered at low risk of developing the disease. However, it has recently been suggested that they should also be considered as affected, as their risk of progression to kidney disease is higher when compared to the healthy population without variations in collagen genes [10]. This approach has opened the possibility of expanding the understanding of the syndrome, leading to a broader comprehension of its spectrum, which may result in known but not directly related phenotypes. 

The literature indicates a notable association between variants in the *COL4A* gene family, typically linked to AS, and the occurrence of cystic kidney disease, sometimes even resembling or co-occurring with ADPKD. Single pathogenic variants in the collagen genes *COL4A* (*COL4A5* in females) can result in mild *COL4A*-related disease phenotypes, where kidney cysts can be present [6,11,12]. While ADPKD is predominantly associated with *PKD1* and *PKD2* gene variants, there are documented cases where individuals with pathogenic variants in *COL4A3*, *COL4A4*, or *COL4A5* also present with renal cysts and even a clinical diagnosis of polycystic kidney disease (PKD) [13].

Thanks to expanded research, some patients who were previously considered non-carriers of genetic diseases have been reclassified into other specific syndromes. Currently, it is recommended that genetic studies be extended in patients with bilateral renal cysts who do not have variations in *PKD1* or *PKD2* to avoid misdiagnosis [6].

Kidney cysts associated with *COL4A* variants may differ from those in typical ADPKD. They tend to be fewer in number, enlarge more slowly, may not significantly increase kidney volume, and are often not accompanied by liver cysts. The absence of extra-renal lesions on ultrasound can suggest *COL4A* defects rather than ADPKD [14]. With the use of next-generation sequencing (NGS), an exciting new perspective has emerged in understanding kidney diseases by identifying known pathogenic genes in diseases that were previously not associated with them [15].

A particularly high prevalence of cystic kidney disease has been observed in patients with AS [16]. Nowadays, renal cystic disease is increasingly believed to be related to AS or a spectrum of it, with the association of both appearing to worsen the prognosis of patients through mechanisms that are not yet fully understood. Unlike that of ADPKD, the pathophysiology of this disease does not seem to be directly related to the size and growth of cysts, which are typically small to medium in size, grow slowly, are bilateral, do not preferentially affect the cortex or medulla, have homogeneous and unorganized content, and do not significantly alter the total kidney size [12]. There are other indications of a probable relationship between *COL4A* variations and PKD. Hematuria is a highly prevalent clinical sign in patients with simple cysts or cysts outside of ADPKD [17]. Kidney manifestation in ADPKD, including hematuria, flank pain, cyst infections, and kidney stones, is closely associated with kidney cyst burden [18]. Given the high prevalence of “non-pathological” microscopic hematuria in patients with *COL4A* variations, as well as its association with the development of cysts that appear to have characteristics of simple cysts, knowledge of the *COL4A* genes in a patient with simple cysts and hematuria of unknown etiology could help avoid unnecessary interventions. 

Interestingly, recent studies show that not all simple cysts are completely benign. It has been observed that individuals with renal cysts are more likely to develop kidney damage, which varies according to the size and growth rate of the cysts. The impact of genetic causes on the totality of PKD is still unclear.

Given the growing evidence about the pathological significance of PKD previously considered simple [19], further investigation of non-syndromic or simple cysts should be promoted, and such cysts should no longer be considered a completely benign entity as previously thought. In this review, we aim to focus on part of the pathophysiology of renal cystic disease, explained through variations in type IV collagen.

## 2. Cysts and *COL4A*: Insights into Molecular Mechanisms

Collagen IV is a fundamental component of basement membranes, providing structural support and participating in cell signaling within various tissues, including the kidney [20].

The collagen IV family exhibits a specific tissue distribution: In the human and rodent kidneys, immunohistochemical studies have shown low-level expression of α1(IV) to α2(IV) in mature GBM, whereas the α3(IV) to α5 (IV) chains are highly expressed [9].

Variations in the genes that encode these α chains—especially *COL4A1*, *COL4A3*, *COL4A4*, and *COL4A5*—have been associated with several kidney disorders, which involve not only glomerular dysfunction but also the formation of cysts.

At a molecular level, variations in *COL4A* genes can disrupt the intricate assembly and stability of the collagen IV network within the basement membrane [21].

These disruptions can compromise the structural integrity of renal tubules and glomeruli, potentially leading to the formation of cysts. For instance, in HANAC (hereditary angiopathy, nephropathy, aneurysms, and muscle cramps) syndrome, caused by *COL4A1* variations, patients often present with multicystic kidneys, suggesting that the pathogenic effects may involve defective cell–basement membrane interactions. The primary alteration involves the α1 chain of type IV collagen (*COL4A1*), and this defect affects the formation and integrity of the collagen IV α1α1α2 network, which is crucial for the early stages of GBM development and persists in the mature kidney. *COL4A1* pathogenic variants specifically disrupt cell–matrix contacts, particularly those mediated by integrins [8].

Altered interactions with other extracellular matrix components due to defective collagen IV could also play a role in cystogenesis [22].

One explanation for the association of *COL4A3*, *COL4A44*, and *COL4A5* variants with kidney cysts is that the cysts result from the distension of basement membranes weakened by disruption of the collagen IV α3α4α5 network. These cysts likely originate from the glomeruli or distal tubules where these collagen chains are expressed [14], but the mechanisms underlying cystogenesis in these *COL4A* variant carriers are not yet fully understood and could potentially be linked to underlying chronic kidney disease (CKD) or other factors; their co-occurrence suggests a more complex interplay between these genes and cystic kidney phenotypes than previously appreciated [13].

The use of animal models, particularly murine models with targeted variations in *COL4A* genes, has provided helpful insights into the pathogenesis of kidney diseases associated with *COL4A* defects, including the development of renal cysts. While in humans, AS, caused by variations in *COL4A3*, *COL4A4*, and *COL4A5*, is primarily considered a glomerulopathy, studies in mouse models have revealed that initial symptoms can appear in the tubule–interstitial region, indicating a broader impact of collagen IV dysfunction. Moreover, the severity of disease in the tubule–interstitial region, including cyst formation, can be influenced by the genetic background of the mouse model [21]. 

Mice carrying the *COL4A1* p. Gly498Val variation, which was identified in a family with HANAC, specifically developed multicystic kidneys, thereby providing a model system to investigate the pathophysiology of renal defects associated with this particular variation [8].

Another study on mice with AS (*COL4A3*-knockout 129 Sv/J model) showed increased levels of two similar molecules, matrix metalloproteinase-9 (Mmp9) and matrix metalloproteinase-12 (Mmp12), in the glomeruli. The increase in Mmp9 seemed linked to the activation of a certain cell pathway (ERK), while the increase in Mmp12 appeared to be related to a specific chemical signal (through the CCR2 receptor) that could be related to cyst formation [23].

Interestingly, studies on Bull Terriers with hereditary nephritis, a model for autosomal dominant AS, have demonstrated the presence of cystic structures in newborn animals; specifically, cortical and medullary tubular dilatation, as well as dilatation of peritubular veins and capillaries, were observed in three out of six affected neonates [24].

## 3. Cysts and *COL4A*: Evidence from Human Studies

A cystic phenotype is increasingly recognized in association with disorders caused by pathogenic variants in the *COL4A3*, *COL4A4*, and *COL4A5* genes; several studies have investigated the prevalence of kidney cysts in individuals with these genetic variants.

Notably, Furlano et al. conducted a large multicenter cohort study demonstrating an increased prevalence of kidney cysts in individuals carrying heterozygous pathogenic/likely pathogenic variants in the *COL4A3* or *COL4A4* genes compared to the general population [25]. They conducted a study on 157 adults with pathogenic or likely pathogenic variants in the *COL4A3* or *COL4A4* genes and found that 53.5% had at least one kidney cyst (1+ KC) in either kidney, and 28.0% had three or more kidney cysts (3+ KCs) in either kidney. The study demonstrated a higher prevalence of kidney cysts in these individuals compared to the general population when matched for sex and age. Furthermore, the presence of kidney cysts, particularly 3+ KCs, was associated with older age and lower estimated glomerular filtration rate (eGFR).

This study clarified that kidney cysts are a more frequent occurrence in individuals with *COL4A3* or *COL4A4* variants than previously acknowledged and stressed the importance of considering AS in the differential diagnosis of hereditary cystic kidney diseases. 

Chang et al. described three cases of children with AS who also presented with kidney cysts. In one case, multiple large cysts were observed in the bilateral medullary area, mimicking ADPKD, while in the other two cases, multiple bilateral medullary cysts developed during the follow-up of children with X-linked AS, sometimes with nephromegaly. Light microscopy in all three cases showed dilatation of the distal tubules [26].

Also, in a study by Teresa Bada-Bosch et al. [12], the finding of 31 autosomal dominant AS patients with *COL4A3* or *COL4A4* variants highlights the common occurrence of multicystic kidney disease (MKD) (52%) and its association with a poorer renal prognosis, as evidenced by a faster eGFR decline, underscoring the clinical relevance of screening for MKD in this population.

Pierides et al. described multiple small and large kidney cysts in four older individuals among 236 family members with a biopsy-proven diagnosis of thin basement membrane disease (TBMD) and microhematuria and/or heterozygous pathogenic COL4A3 variants [27]. 

Another study by Sevillano et al. [28] focused on patients with biopsy-proven TBMD, which is often caused by heterozygous *COL4A3* or *COL4A4* variations. In this cohort of 32 patients, bilateral kidney cysts were found in 56% (9 out of 16) of patients who also had proteinuria and chronic kidney disease (CKD). Notably, no cysts were found in the 16 patients with TBMD who had persistent normal kidney function and negative or minimal proteinuria. 

Another case was reported by Savige et al., who confirmed X-linked AS in a male patient with uremia and bilateral kidney cysts of unclear etiology [29].

Variations in *COL4A1* have been associated with HANAC (hereditary angiopathy, nephropathy, aneurysms, and muscle cramps) syndrome, which can present nephropathy and renal cysts. Specifically, patients with this variation exhibit severe ultrastructural defects in the basement membrane of Bowman’s capsules, tubules, and interstitial capillaries. In other cases, large renal cysts have been observed, particularly in the renal poles, although the overall kidney size remains relatively unaffected [30].

A study by Gulati et al. [6] investigated adults with bilateral renal cysts and CKD who were negative for *PKD1* and *PKD2* variations. While the primary focus was on identifying *COL4A1* variations, the study also included one patient with TBM disease and bilateral renal cysts who was heterozygous for a likely pathogenic missense variant in *COL4A5*.

A study by Izzi et al. [16] specifically investigated the cystic kidney phenotype in a cohort of 108 patients diagnosed with AS. They found that 42% (33 out of 79) of patients with available renal imaging had kidney cysts. According to this study, it is hypothesized that pathogenic variants in *COL4A3*/*COL4A5* that disrupt the type IV collagen α3α4α5 network may result in weakened basement membranes in the distal tubule, making them more susceptible to cyst formation.

Finally, it is important to note that the KDIGO (Kidney Disease Improving Global Outcomes) 2025 ADPKD Guidelines indicate that pathogenic variants in the collagen genes *COL4A3*, *COL4A4*, and *COL4A5* may be associated with mild phenotypes of type IV collagen-related disease, in which renal cysts can be observed. Furthermore, the 2024 guidelines on behalf of ERKNet, ERA, and ESPN, focusing on disorders resulting from genetic defects in the *COL4A3*/*4*/*5* genes (Alport syndrome spectrum disorders), explicitly include cystic kidney disease as a criterion for which genetic testing for these genes should be performed. Notably, *COL4A3* and *COL4A4* are specifically mentioned as genes to be included in cystic kidney disease panels [31].

## 4. Cysts and *COL4A*: Co-Occurrence with ADPKD Gene Variants

The occurrence of PKD as a clinical manifestation in patients with *COL4A3-A5* variants is gaining increasing attention. [6] investigated collagen IV gene variants in adults with bilateral renal cysts and CKD, highlighting a potential link between *COL4A* pathogenic variants and cystic kidney disease. Additionally, [29] stated that AS with kidney cysts is still AS. [16] further explored the clinical implications and significance of the cystic phenotype in AS. 

Given the high frequency of pathogenic or likely pathogenic variants in *COL4A3* and *COL4A4*, the potential co-occurrence of these pathogenic variants with PKD-related gene variants (*PKD1*, *PKD2*) warrants closer investigation. Notably, the absence of extra-renal lesions on ultrasound may point toward *COL4* defects rather than ADPKD [14]. 

However, differential diagnosis can be challenging. For instance, a recent case report [13] describes a 24-year-old male proband with early-onset PKD, sensorineural hearing loss, and persistent hematuria who was found to have a novel frameshift variant in *COL4A5*. Interestingly, the proband’s mother, a heterozygous carrier of the same *COL4A5* variant, presented PKD, IgA glomerulonephritis, and focal segmental glomerulosclerosis (FSGS). This case supports the emerging association between PKD and collagen type IV defects.

Another study [32] described a patient with pathogenic variants in both *PKD1* (c.11648_11660dup; p. Phe3888fs) and *COL4A4* (c.3418_3424del; p. Leu1140fs), leading to diagnoses of both ADPKD and AS. These findings suggest that the co-inheritance of pathogenic or likely pathogenic variants in these distinct sets of genes can occur and may contribute to complex clinical presentations [32].

Further genetic evidence comes from a study utilizing a next-generation sequencing (NGS) panel for hereditary kidney diseases, which identified pathogenic or likely pathogenic variants in 108 patients [15]. Among these, 55 variants were in genes associated with AS (*COL4A3*, *COL4A4*, *COL4A5*) and 47 were in genes associated with ADPKD (*PKD1*, *PKD2*). Although this study did not explicitly detail the co-occurrence of variants in both sets of genes within the same individuals in their main findings, it underscored the frequency of variants in these genes in a cohort with suspected hereditary kidney diseases [13,14]. Overall, these findings underscore the importance of comprehensive genetic screening in patients with cystic kidney disease, particularly when clinical features overlap with those of Alport syndrome or ADPKD.

## 5. Discussion

We highlighted how genetic alterations in *COL4A* genes in mice have been extensively implicated in the development of renal cystic phenotypes, with AS serving as a primary model. While AS in humans is characterized by a phenotype of microhematuria, deafness, and visual impairment due to glomerulopathy, murine models reveal that the pathology can manifest at the tubulointerstitial level, suggesting a role for the microenvironment and extracellular matrix composition triggered by the loss of collagen α3/α4(IV). It is crucial to note how collagen genetics influence glomerular extracellular matrix composition and organization, modulating the progression of cystic disease. The demonstration of an isoform switch towards collagen α5α6(IV) in certain *COL4A3*-/- models associated with increased renal survival underscores the plasticity and compensatory mechanisms activated in response to collagen IV deficiency [21].

In the context of translational research, the observation of renal cysts in human patients with collagen IV gene variations, even without concurrent *PKD1* or *PKD2* variations, has opened new avenues for understanding the pathogenesis of renal cystic diseases. The identification of variants in *COL4A3*, *COL4A4*, *COL4A5*, and *COL4A1* in individuals displaying cystic phenotypes and chronic kidney disease (CKD) suggests that the structural and functional compromise of renal basement membranes, stemming from collagen IV variations, may predispose such individuals to cystogenesis. Notably, missense variants affecting glycine residues are significantly more frequent in patients with a cystic phenotype compared to truncating variants. This finding hints at a potential genotype–phenotype correlation concerning cyst development in AS [16].

The hypothesis that pathogenic variants in *COL4A3-COL4A5*, compromising the α3α4α5(IV) collagen network, may weaken basement membranes in the distal tubule, rendering them more susceptible to cyst development, is supported by animal studies. Indeed, Zeni et al. postulated that pathogenic variants in *COL4A3*/*COL4A5* disrupting the α3α4α5(IV) collagen network can result in weakened basement membranes in the distal tubule, increasing their vulnerability to cyst formation. It is suggested that dysfunction of a single gene encoding collagen IV isoforms can lead to a reduced quantity of collagen along glomerular or tubular basement membranes and the formation of cysts of glomerular or tubular origin. Experimental studies in animals with variations in various genes encoding collagen IV proteins have demonstrated the onset of glomerulocystic disease and cystic dilation of Bowman’s capsule and tubules [13,26].

The occurrence of renal cysts in patients with *COL4A3-COL4A5* variants, independently of *PKD1* or *PKD2* alterations, underscores the broader implications of collagen IV defects in cystogenesis. Similarly, pathogenic variants in *COL4A1* further emphasize the connection between basement membrane integrity and cyst formation, affecting the α1α1α2(IV) network predominantly in Bowman’s capsule and tubules. Pathogenic variants in *COL4A1* primarily affect the α1α1α2(IV) network, leading to structural defects in basement membranes of Bowman’s capsule, tubules, and interstitial capillaries. HANAC syndrome, caused by *COL4A1* variants, further highlights the role of collagen IV in maintaining renal architecture and preventing cyst formation. Despite these abnormalities, the GBM typically remains unaffected due to its predominant α3α4α5(IV) composition [30].

Despite these advances, several open questions remain. The precise molecular and cellular mechanisms through which collagen IV variations lead to cyst formation require further elucidation. It is crucial to understand whether cystogenesis is a direct consequence of basement membrane structural fragility, leading to tubular dilation, or if more active mechanisms are involved, such as altered cell polarity, abnormal epithelial proliferation, or the activation of specific signaling pathways. Future studies should prioritize the investigation of cell–matrix interactions in renal cystogenesis, particularly focusing on collagen IV receptors such as integrins and discoidin domain receptors. Additionally, exploring the activation of the unfolded protein response in response to collagen IV deficiency may provide insights into the molecular mechanisms underlying cyst formation. Finally, it is crucial to define more precisely the prevalence and clinical significance of the cystic phenotype in patients with AS and to evaluate whether the presence of cysts can influence the progression of kidney disease and the response to therapies. 

Considering the relatively high population frequency of variants in collagen genes (1:106), potential overlap between genetic and acquired renal phenotypes is plausible. As highlighted in the provided information, instances exist where individuals present clinical features suggestive of PKD alongside identified variants in *COL4A3*, *COL4A4*, or *COL4A5* [13,32]. In such cases, it is not entirely possible to exclude the co-inheritance of pathogenic or likely pathogenic variants in genes typically associated with ADPKD, such as *PKD1* and *PKD2*, or less common ADPKD-related genes like *GANAB* and *DNAJB11* [31].

The current diagnostic approach often involves targeted genetic testing based on the primary clinical suspicion. Consequently, when a variant is identified in a collagen gene in a patient with cystic kidney features, a comprehensive evaluation for concurrent variants in *PKD1*, *PKD2*, or other cystic kidney disease-associated genes might not be routinely performed [15,33]. As suggested, a study specifically designed to assess the prevalence of co-occurring pathogenic or likely pathogenic variants in both collagen genes (*COL4A3*, *COL4A4*, *COL4A5*) and ADPKD-related genes (*PKD1*, *PKD2*, *GANAB*, *DNAJB11*) within the same individuals would be valuable. Such an investigation could elucidate the true extent of genetic overlap and its contribution to the observed phenotypic complexity in patients with renal cysts and other associated features [13,32]. This is particularly relevant given the emerging recognition of PKD-like manifestations in individuals with *COL4A3-A5* variants [6,14].

Despite this evidence, the AS guideline [31] does not describe renal cysts as a typical or prominent feature of AS, focusing primarily on glomerular manifestations. However, it acknowledges that AS can be diagnosed following genetic testing in patients with various renal disease presentations, including cysts.

Integrating genetic, radiological, and histopathological data from large patient cohorts will be essential to delineate the role of collagen IV variations in the spectrum of renal cystic diseases and to improve the diagnosis and clinical management of these conditions.

Therefore, renal cyst formation in patients with *COL4A* variations appears to stem from widespread structural compromise of basement membranes, affecting both glomerular and tubular compartments. Clinically, this highlights the importance of recognizing cystic phenotypes as part of the spectrum of collagen IV nephropathies. Mechanistically, further studies are needed to elucidate whether cystogenesis is primarily driven by basement membrane fragility or involves additional processes such as epithelial proliferation and aberrant signaling pathways.

## 6. Conclusions

In conclusion, accumulating evidence from the recent literature and specific studies on the cystic phenotype in AS increasingly supports the correlation between variants of collagen IV genes and the propensity for developing renal cysts, likely mediated through mechanisms affecting the structural integrity of renal basement membranes. New guidelines [4] for the evaluation, management, and treatment of ADPKD have emphasized the key features that help distinguish ADPKD from other cystic renal disorders with similar clinical presentations. These include certain collagen-related diseases, often characterized by hematuria, and structural abnormalities of the glomerular basement membrane. Furthermore, the available evidence indicates that renal cysts can indeed be a feature in individuals harboring variants in *COL4A3*, *COL4A4*, and *COL4A5*. Notably, documented cases exist where individuals with these *COL4A* variants also present with clinical features or a diagnosis of PKD, sometimes co-occurring with variants in *PKD1* or *PKD2*. Consequently, comprehensive kidney disease gene panel testing is crucial for achieving accurate diagnoses in such complex clinical presentations.

## Figures and Tables

**Table 1:** Comparison between autosomal dominant polycystic kidney disease (ADPKD) and Alport syndrome (AS) with respect to genetic cause, cyst prevalence and characteristics, and kidney size.

	Autosomal Dominant Polycystic Kidney Disease (ADPKD)	Alport Syndrome (AS)
**Genetic Cause**	Primarily heterozygous pathogenic variants in the *PKD1* and *PKD2* genes.	Pathogenic variants in genes encoding collagen IV: *COL4A3*, *COL4A4*, and *COL4A5*. Digenic inheritance can also occur.
**Cyst Prevalence**	Cysts are a hallmark feature, and diagnosis is typically based on imaging criteria showing multiple cysts.	A cystic phenotype has been found in a significant proportion of patients.
**Cyst Characteristics**	Typically, numerous and large. The expansion of cysts in ADPKD may play a role in the increase in arterial pressure.	Consist of multiple bilateral cortical kidney cysts. They are typically less numerous and smaller than cysts in ADPKD. The presence of cysts is a distinct phenotype in Alport syndrome.
**Kidney Size**	Kidneys are typically enlarged. The Total Kidney Volume (TKV) is a prognostic marker. There is a strong relationship between kidney volume and adverse outcomes.	Most patients with autosomal dominant Alport syndrome (ADAS) have normal or reduced kidney size. However, in rare cases with severe cysts, a notable increase in TKV that mimics ADPKD can be observed. Kidney length and volume can be measured by magnetic resonance imaging and ultrasound.

## Data Availability

All data discussed in this review are in the public domain.

## Abbreviations

**AD**, autosomal dominant; **ADPKD**, autosomal dominant polycystic kidney disease; **AS**, Alport syndrome; **CKD**, chronic kidney disease; **eGFR**, estimated glomerular filtration rate; **ERA**, European Renal Association; **ERKNet**, European Reference Network on Rare Kidney Diseases; **ESPN**, European Society for Paediatric Nephrology; **FSGS**, focal segmental glomerulosclerosis; **GBM**, glomerular basement membrane; **HANAC**, hereditary angiopathy, nephropathy, aneurysms, and muscle cramps; **KC**, kidney cyst; **KDIGO**, Kidney Disease Improving Global Outcomes; **MKD**, multicystic kidney disease; **mmp9**, matrix metalloproteinase-9; **mmp12**, matrix metalloproteinase-12; **NGS**, next-generation sequencing; **PKD**, polycystic kidney disease; **TBM**, tubular basement membrane; **TBMD**, thin basement membrane disease.
